# Calcium Channel Subunit Alpha2delta 2 Is Upregulated During Odontoblast-Like Differentiation and Exhibits Higher Basal Expression in Stem Cells From Exfoliated Deciduous Teeth Than in Dental Pulp Stem Cells

**DOI:** 10.7759/cureus.113676

**Published:** 2026-07-30

**Authors:** Fidele Nabbout, Michella Ghassibe, Joseph Sabbagh

**Affiliations:** 1 Department of Orthodontics, Faculty of Dental Medicine, Lebanese University, Beirut, LBN; 2 Department of Natural Sciences, School of Arts and Sciences, Lebanese American University, Beirut, LBN; 3 Department of Restorative Dentistry and Endodontics, Lebanese University, Beirut, LBN

**Keywords:** cacna2d2, calcium channel, dental pulp stem cells, dentinogenesis, odontoblast differentiation, regenerative dentistry, rt-pcr, shed, trio

## Abstract

Introduction: Recent work in Lebanese families with familial non‑syndromic tooth agenesis identified calcium voltage‑gated channel auxiliary subunit alpha2delta 2 (CACNA2D2) and triple functional domain protein (TRIO) as candidate genes, and a subsequent computational study linked both to an odontogenesis‑relevant signaling framework. However, it remains unclear whether CACNA2D2 and TRIO are expressed in human dental stem cells and whether their transcript levels change during odontogenic induction. Materials and methods: Dental pulp stem cells (DPSCs) and stem cells from human exfoliated deciduous teeth (SHED) were cultured under standard mesenchymal stem cell conditions, and DPSCs were exposed to odontogenic medium for 21 days to generate odontoblast‑like cells. Total ribonucleic acid (RNA) was extracted from undifferentiated DPSCs, differentiated odontoblast‑like cells, and undifferentiated SHED, and CACNA2D2 and TRIO expression was evaluated by semi‑quantitative reverse transcription polymerase chain reaction (RT‑PCR) with glyceraldehyde‑3‑phosphate dehydrogenase (GAPDH) normalization and ImageJ‑based densitometry in five independent biological replicates. Pairwise group comparisons were analyzed using the two‑tailed Student’s t‑test. Results: CACNA2D2 expression was higher in odontoblast‑like cells than in undifferentiated DPSCs, with semi‑quantitative densitometry indicating an approximate threefold increase (two‑tailed Student’s t‑test, t(8) = 3.21, p = 0.016). Basal CACNA2D2 expression was also higher in SHED than in DPSCs, with an approximate sevenfold difference (t(8) = 4.50, p = 0.001). In contrast, TRIO expression did not differ significantly between groups (t(8) = 0.84, p = 0.42, and t(8) = 0.67, p = 0.52). Conclusions: CACNA2D2, but not TRIO, showed differentiation‑associated and cell‑type‑dependent transcriptional variation in human dental pulp‑derived stem cell models under odontogenic induction conditions. These findings provide preliminary semi‑quantitative wet‑laboratory support for CACNA2D2 as a gene of interest in human odontogenesis and familial tooth agenesis but remain correlative and should be confirmed by quantitative reverse transcription polymerase chain reaction (RT‑qPCR), canonical odontogenic differentiation markers, mineralization assays, and functional studies in the same experimental system.

## Introduction

Tooth agenesis is a frequent developmental abnormality in which one or more teeth fail to form, reflecting disruption of the coordinated molecular events that guide tooth initiation, morphogenesis, and terminal differentiation. Although established genes explain part of the disease burden, many familial cases remain incompletely understood, which suggests that additional regulators of odontogenesis remain to be characterized [1].

Two recent studies from the same research context brought attention to the calcium voltage‑gated channel auxiliary subunit alpha2delta 2 (CACNA2D2) and the triple functional domain protein (TRIO) as candidate contributors to familial non-syndromic tooth agenesis in Lebanese families. The first study used clinical characterization and whole-exome sequencing to identify rare segregating variants in these genes in two multigenerational families. The second study extended those observations through domain mapping, conservation analysis, structural inspection, and interaction-network modeling. It proposed a TRIO-Ras homolog family member A (RHOA)-G protein subunit beta 1 (GNB1)-CACNA2D2 axis as a testable mechanistic framework in odontogenesis [2,3]. Importantly, both studies were hypothesis-generating and did not include direct expression or functional experiments in dental stem cell systems.

This unresolved gap is relevant because odontogenesis depends not only on developmental patterning pathways but also on the cellular machinery required for polarization, secretion, matrix deposition, and mineralization [4,5]. Calcium handling is particularly important during odontoblast differentiation because dentin-forming cells must coordinate ion transport with extracellular matrix production and mineral deposition. CACNA2D2 encodes the alpha-2/delta-2 auxiliary subunit of voltage-gated calcium channels, a protein known to influence channel trafficking, membrane localization, and current properties [4,5]. On biologic grounds alone, this makes CACNA2D2 a plausible gene to examine in dental stem cells undergoing odontogenic commitment.

TRIO is a second plausible candidate because it encodes a multidomain Rho guanine nucleotide exchange factor involved in cytoskeletal regulation, cell polarity, and migratory behavior [6,7]. These processes are integral to the organization of craniofacial tissues and to the acquisition of the polarized secretory phenotype typical of mature odontoblasts [7-9]. Even if TRIO transcript abundance does not change markedly during differentiation, its baseline expression in relevant dental cell types remains worth establishing experimentally.

Among available in vitro models, dental pulp stem cells (DPSCs) and stem cells from human exfoliated deciduous teeth (SHED) are particularly useful because both are accessible dental mesenchymal stem cell populations with recognized odontogenic potential. At the same time, they are not biologically identical; prior work has suggested differences in proliferative behavior, mineralization tendencies, and lineage-related characteristics between stem cells derived from permanent and deciduous teeth. A comparison between these cell types may reveal whether candidate odontogenesis-related genes are broadly shared or exhibit stem-cell-type-specific expression patterns [10-14].

The present study was designed as a focused wet-laboratory extension of the earlier clinical-genetic and in silico observations. It aimed to determine whether CACNA2D2 and TRIO are expressed in human dental pulp-derived stem cell models, whether CACNA2D2 or TRIO transcription changes during odontoblast-like differentiation of DPSCs, and whether baseline expression differs between DPSCs and SHED. Quantitative gene expression analyses were conducted in accordance with current quantitative reverse transcription polymerase chain reaction (RT-qPCR) methodological and reporting standards [15]. By addressing these questions, this study does not attempt to establish pathogenicity or define a mechanism but rather to establish a first experimental expression-level link between these candidate genes and a relevant human odontogenic cell context.

Objectives

The objective of this study was to evaluate the expression of CACNA2D2 and TRIO in human DPSC models and to explore their potential relevance to odontogenic differentiation. Specifically, we aimed to (1) assess baseline expression of both genes in DPSCs and SHED, (2) examine changes in CACNA2D2 and TRIO transcription during odontoblast-like differentiation of DPSCs, and (3) compare expression patterns between these stem cell populations to identify potential cell-type-specific differences related to odontogenesis.

## Materials and methods

Ethical approval and cell sources

Human dental pulp-derived cells used in this study were obtained from healthy donors who provided written informed consent for the use of their tissues in research. All procedures were conducted in accordance with the Declaration of Helsinki and were approved by the Institutional Review Board of Lebanese American University (approval number: LAU.SAS.MS1/23/May/2018, approval date: May 23, 2018). DPSCs and SHED were used as the cellular models for all gene expression experiments performed in this study.

Cell culture conditions

DPSCs and SHED were maintained in Dulbecco's Modified Eagle Medium/F-12 (DMEM/F-12) supplemented with 10% fetal bovine serum (FBS) at 37°C in a humidified atmosphere containing 5% carbon dioxide. Cells were passaged at approximately 80% confluence and were used between passages 3 and 5 to reduce variability related to prolonged in vitro expansion.

Odontogenic induction of DPSCs

For differentiation experiments, DPSCs were seeded at 1 × 10⁴ cells/cm² and allowed to reach approximately 70% confluence before exposure to odontogenic medium. The induction medium consisted of minimum essential medium alpha (α-MEM) supplemented with 10⁻⁴ M L-ascorbic acid 2-phosphate, 10⁻⁸ M dexamethasone, 5 mM beta-glycerophosphate, and 1.8 mM monopotassium phosphate. Medium was changed every two to three days, and cells were harvested after 21 days of induction. In this context, we considered the induced DPSCs as “odontoblast-like” based on the odontogenic medium and previous work using similar protocols to drive odontogenic commitment. However, we did not measure dentin-related markers such as dentin sialophosphoprotein (DSPP), dentin matrix acidic phosphoprotein 1 (DMP1), alkaline phosphatase (ALP), or Runt-related transcription factor 2 (RUNX2), nor did we perform Alizarin Red S mineralization assays in the same cultures, which are commonly used to validate odontoblastic differentiation. For this reason, we deliberately use the term “odontoblast-like cells” throughout and do not claim full phenotypic confirmation of an odontoblast identity. Although this odontogenic medium and induction protocol have been widely used to promote odontogenic commitment of DPSCs in previous studies, in the present work, we did not perform additional phenotypic validation (e.g., assessment of dentin‑related markers such as DSPP, DMP1, ALP, RUNX2, or Alizarin Red S mineralization assays). Therefore, we deliberately use the term “odontoblast‑like cells” without claiming full odontoblast identity.

RNA extraction and reverse transcription

Total ribonucleic acid (RNA) was isolated from undifferentiated DPSCs, 21‑day odontoblast-like cells, and undifferentiated SHED using TRIzol reagent according to the manufacturer's instructions. RNA quality and concentration were evaluated spectrophotometrically, using A260/280 values of at least 1.8 and A260/230 values of at least 1.7 as quality thresholds. First‑strand complementary deoxyribonucleic acid (cDNA) was synthesized from 1 microgram of total RNA using the iScript First Strand cDNA Synthesis Kit with random hexamer primers.

PCR amplification and densitometric analysis

PCR was performed according to the manufacturer’s recommended protocol for the Thermo PCR Reaction Mix Kit (Thermo Fisher Scientific, Waltham, MA, USA), using 35 cycles with gene‑specific annealing temperatures summarized in Table 1. Primer sets targeting CACNA2D2, TRIO, and glyceraldehyde‑3‑phosphate dehydrogenase (GAPDH) were used; all primer sequences, expected amplicon sizes, and annealing temperatures are summarized in Table 1.

**Table 1:** PCR primer sequences Primer sequences used for RT-PCR amplification of CACNA2D2, TRIO, and the housekeeping gene GAPDH. All primers were designed to span exon-exon junctions to minimize amplification of genomic DNA. PCR: polymerase chain reaction, RT-PCR: reverse transcription polymerase chain reaction, CACNA2D2: calcium voltage-gated channel auxiliary subunit alpha2delta 2, TRIO: triple functional domain protein, GAPDH: glyceraldehyde-3-phosphate dehydrogenase, DNA: deoxyribonucleic acid

Gene	Primer	Sequence (5'→3')	Amplicon size	Annealing Tm
CACNA2D2	Forward	AATCGCAGGTGCTTGCTG	~320 bp	58°C
CACNA2D2	Reverse	CCTCACTCCTTATTTCTGAA		
TRIO	Forward	GCCCTGAACACAACACCTTG	~280 bp	60°C
TRIO	Reverse	TAGGACCCTCAGGCTGGC		
GAPDH	Forward	GGATTTGGTCGTATTGGG	~180 bp	56°C
GAPDH	Reverse	GGAAGATGGTGATGGGATT		

PCR products were separated on 1.2% agarose gels stained with SYBR Safe (Thermo Fisher Scientific, Waltham, MA, USA) and imaged using the ChemiDoc Imaging System (Bio-Rad Laboratories, Hercules, CA, USA). Gene expression was assessed by semi-quantitative reverse transcription polymerase chain reaction (RT-PCR) rather than RT-qPCR because the primary aim was to obtain a first-pass, band-intensity-based overview of CACNA2D2 and TRIO transcript behavior under odontogenic induction, rather than absolute quantification, which RT-qPCR more appropriately addresses in accordance with established methodological standards. To maximize reliability within this framework, we empirically verified that 35 PCR cycles remained within the linear amplification range for CACNA2D2, TRIO, and GAPDH, ran all reactions to be compared in parallel on the same gels, and quantified band intensity with ImageJ (National Institutes of Health, Bethesda, MD, USA), normalizing target‑gene signals to GAPDH across five independent biological replicates. This design reduces technical variability and allows detection of robust fold‑direction and approximate fold‑magnitude differences, even though it does not provide the dynamic range and precision of RT‑qPCR.

Statistical analysis

Statistical analyses were performed using SPSS Statistics version 28.0 (IBM Corp. Released 2021. IBM SPSS Statistics for Windows, Version 28.0. Armonk, NY). All experiments were performed using five independent biological replicates. Data are presented as mean ± standard deviation (SD), and pairwise comparisons were analyzed using the two‑tailed Student’s t‑test. The significance level was set at 0.05, and a p-value less than 0.05 was considered statistically significant.

## Results

Three principal observations emerged from the study: CACNA2D2 increased during odontoblast‑like differentiation of DPSCs; baseline CACNA2D2 expression was higher in SHED than in DPSCs; and TRIO transcript abundance remained unchanged across all tested conditions.

CACNA2D2 is upregulated during odontoblast‑like differentiation

**Figure 1:**
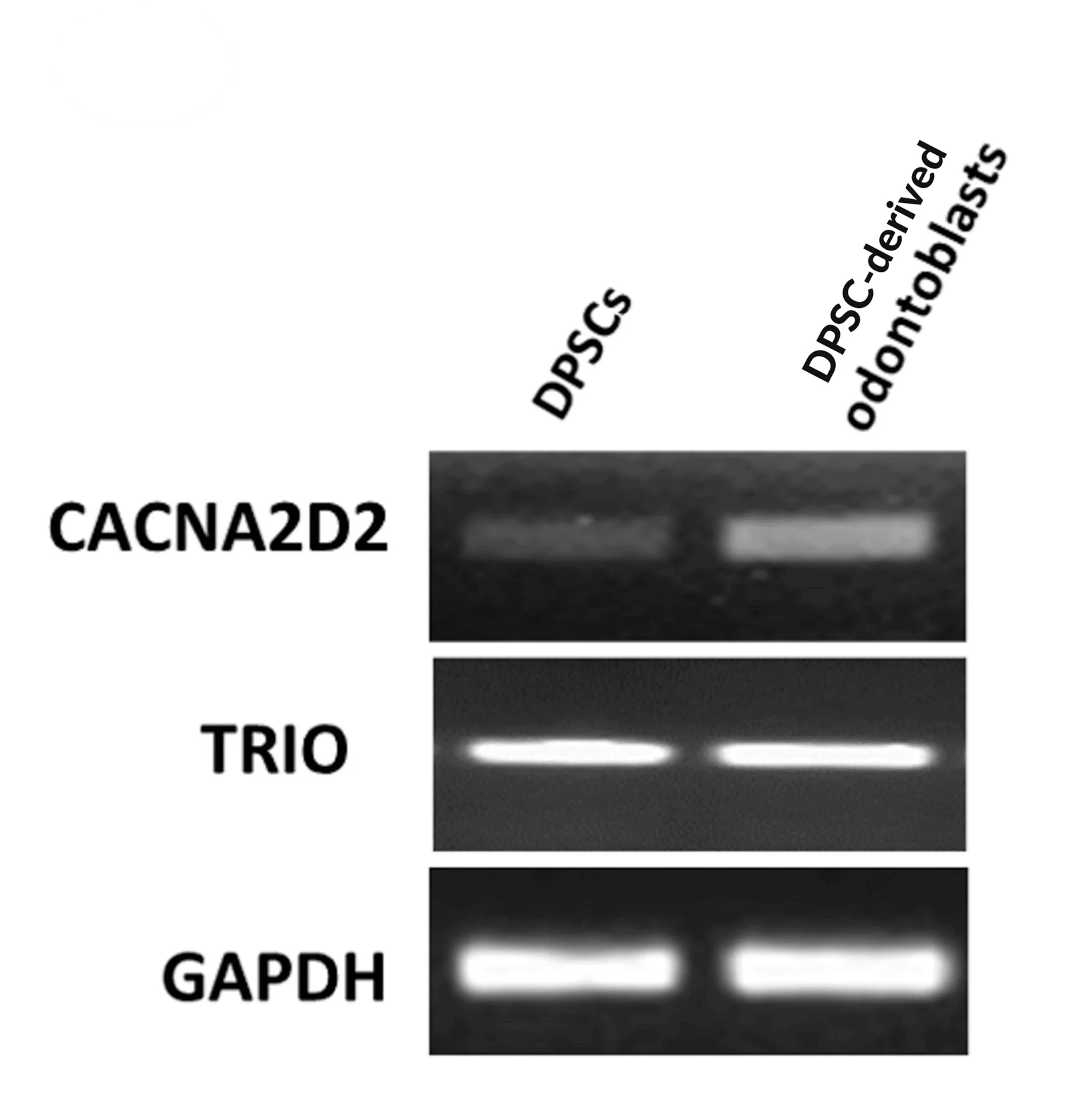
RT-PCR analysis of CACNA2D2, TRIO, and GAPDH expression in undifferentiated DPSCs and DPSC-derived odontoblasts Representative agarose gel showing RT-PCR products for CACNA2D2, TRIO, and GAPDH in undifferentiated DPSCs (left lane) and DPSC-derived odontoblasts at day 21 of odontogenic differentiation (right lane). A visible increase in CACNA2D2 band intensity is observed in the odontoblast condition relative to undifferentiated DPSCs, consistent with transcriptional upregulation during differentiation. TRIO and GAPDH display comparable band intensities across both conditions, confirming constitutive TRIO expression and equal RNA loading. PCR was performed for 35 cycles; amplicons were resolved on a 1.2% agarose gel stained with SYBR Safe and imaged using the ChemiDoc Imaging System. The final figure layout was assembled using Inkscape vector graphics software (Inkscape Project, open-source software). RT-PCR: reverse transcription polymerase chain reaction, CACNA2D2: calcium voltage-gated channel auxiliary subunit alpha2delta 2, TRIO: triple functional domain protein, GAPDH: glyceraldehyde-3-phosphate dehydrogenase, DPSCs: dental pulp stem cells, RNA: ribonucleic acid

Within the limits of this semi‑quantitative RT‑PCR approach, CACNA2D2 band intensity was consistently higher in 21‑day odontoblast‑like cells than in undifferentiated DPSCs across five biological replicates, and representative gels illustrate stronger CACNA2D2 bands in odontoblast‑like cells. In contrast, TRIO and GAPDH bands appear similar between conditions (Figure 1).

**Figure 2:**
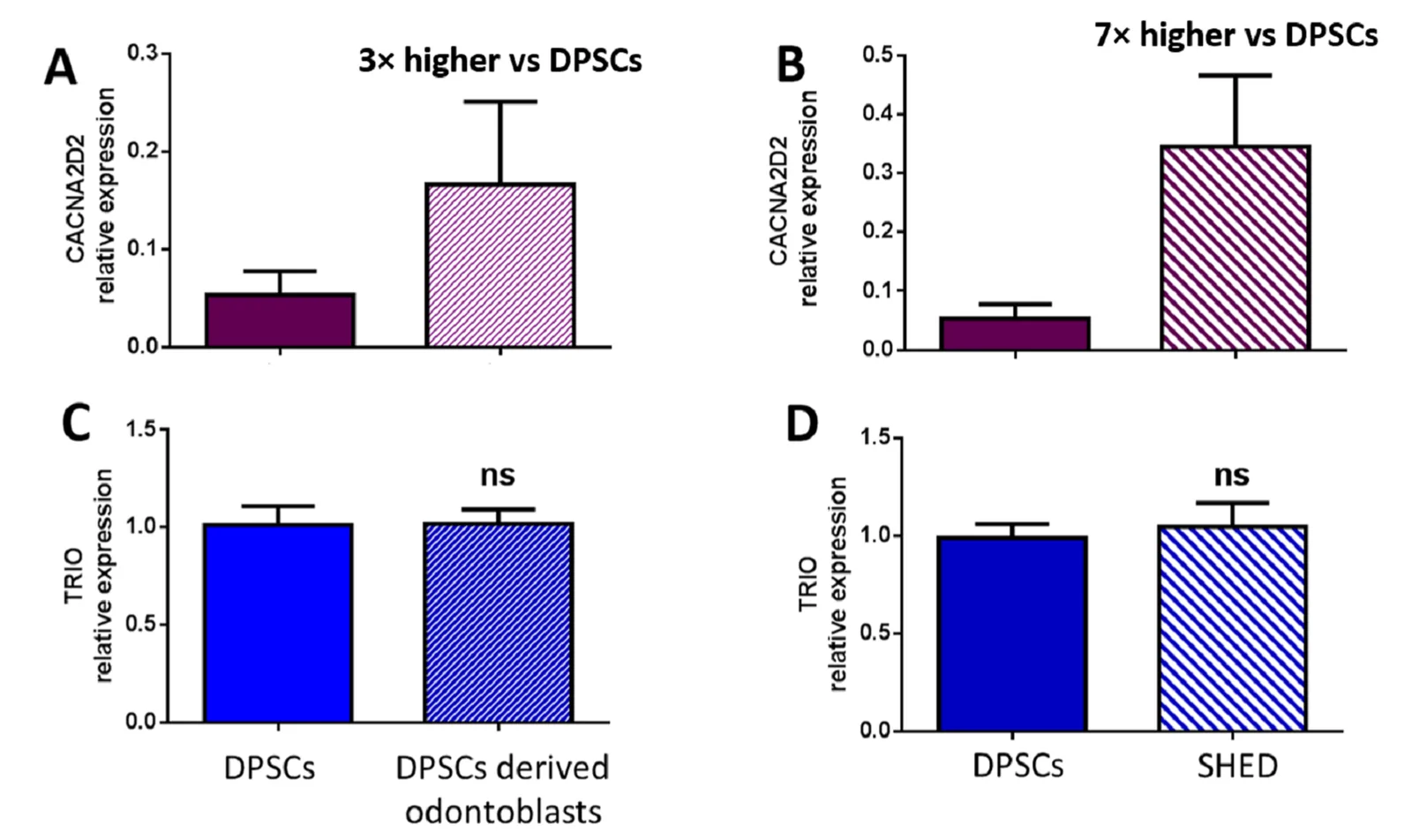
Semi-quantitative RT-PCR expression analysis of CACNA2D2 and TRIO in DPSCs, SHED, and DPSC-derived odontoblasts Bar graphs show relative expression levels of CACNA2D2 (panels A-B) and TRIO (panels C-D), normalized to GAPDH, from five independent biological replicates (n = 5). Data are presented as mean ± SD. Statistical comparisons were performed using two-tailed Student's t-tests. In panel A, CACNA2D2 expression is significantly upregulated by approximately threefold in DPSC-derived odontoblast-like cells at day 21 compared with undifferentiated DPSC controls (t(8) = 3.21, p = 0.016; above the odontoblast-like bar). In panel B, basal CACNA2D2 expression is approximately sevenfold higher in SHED than in undifferentiated DPSCs (t(8) = 4.50, p = 0.001; above the SHED bar). In panel C, TRIO expression shows no significant difference between undifferentiated DPSCs and DPSC-derived odontoblast-like cells (t(8) = 0.84, p = 0.42; ns). In panel D, TRIO expression shows no significant difference between DPSCs and SHED (t(8) = 0.67, p = 0.52; ns). Significance codes: p < 0.05, p < 0.01, and ns, not significant. RT-PCR: reverse transcription polymerase chain reaction, CACNA2D2: calcium voltage-gated channel auxiliary subunit alpha2delta 2, TRIO: triple functional domain protein, GAPDH: glyceraldehyde-3-phosphate dehydrogenase, DPSCs: dental pulp stem cells, SHED: stem cells from human exfoliated deciduous teeth

Densitometric analysis indicated an approximate threefold increase in CACNA2D2 expression in odontoblast‑like cells compared with DPSCs (two‑tailed Student’s t‑test, t(8) = 3.21, p = 0.016; Figure 2A). In contrast, TRIO relative expression did not differ significantly between these groups (two‑tailed Student’s t‑test, t(8) = 0.84, p = 0.42; Figure 2C).

Gene expression differences between groups were evaluated using two‑tailed Student’s t‑tests (n = 5 per group). For each comparison, the corresponding t statistic, degrees of freedom, and p‑value are reported in the Results. Among the evaluated comparisons, CACNA2D2 upregulation during odontoblast‑like differentiation represented the clearest differentiation‑associated change at the transcript level, and the semi‑quantitative RT‑PCR fold changes for CACNA2D2 and TRIO, together with their significance indicators, are summarized in Table 2.

**Table 2:** Summary of expression results Summary of semi-quantitative RT-PCR expression changes for CACNA2D2 and TRIO from five independent biological replicates (n = 5). Values are normalized to GAPDH, and fold changes are calculated relative to undifferentiated DPSCs. An upward arrow (↑) indicates upregulation; ns, not significant; **p < 0.05; *p < 0.01. RT-PCR: reverse transcription polymerase chain reaction, CACNA2D2: calcium voltage-gated channel auxiliary subunit alpha2delta 2, TRIO: triple functional domain protein, GAPDH: glyceraldehyde-3-phosphate dehydrogenase, DPSCs: dental pulp stem cells, SHED: stem cells from human exfoliated deciduous teeth

Gene	Comparison	Fold change	Significance symbol
CACNA2D2	DPSCs vs. DPSC‑derived odontoblasts	Approximately threefold ↑ in odontoblasts	*
CACNA2D2	DPSCs vs. SHED	Approximately sevenfold ↑ in SHED	**
TRIO	DPSCs vs. DPSC‑derived odontoblasts	Approximately onefold (no change)	ns
TRIO	DPSCs vs. SHED	Approximately onefold (no change)	ns

SHED exhibits higher basal CACNA2D2 expression than DPSCs. Present RT‑PCR expression data and key literature comparisons between DPSCs and SHED relevant to odontoblast differentiation are summarized in Table 3. SHED also exhibited markedly stronger CACNA2D2 bands than DPSCs, corresponding to an estimated severalfold higher basal CACNA2D2 expression when normalized to GAPDH (approximately sevenfold; two‑tailed Student’s t‑test, t(8) = 4.50, p = 0.001; Figure 2B). By contrast, TRIO relative expression remained statistically unchanged between SHED and DPSCs (two‑tailed Student’s t‑test, t(8) = 0.67, p = 0.52; Figure 2D). Given the semi‑quantitative nature of RT‑PCR, these findings should be interpreted as pattern‑level evidence of basal enrichment rather than precise quantitative effect sizes. Still, they suggest that CACNA2D2 may help distinguish, at least in part, between stem cell populations derived from deciduous and permanent dental tissues. These observations motivate future RT‑qPCR and functional studies for more rigorous quantitative confirmation. A broader overview of DPSCs and SHED, integrating the present CACNA2D2/TRIO expression patterns with literature‑based differences in proliferation, ALP activity, odontogenic marker expression, mineralization potential, and proposed regenerative advantage, is presented in Table 3 [12-22].

**Table 3:** Comparative molecular and functional profile of DPSCs and SHED relevant to odontoblast differentiation Comparative molecular and functional profile of DPSCs and SHED relevant to odontoblast differentiation. Present study expression data are derived from semi-quantitative RT-PCR normalized to GAPDH and expressed relative to DPSCs, showing higher basal CACNA2D2 expression in SHED (approximately sevenfold; p = 0.001) and no difference in TRIO expression between the two cell populations (ns). Literature-based comparisons of proliferation, ALP activity, odontogenic marker expression, mineralization capacity, and proposed regenerative advantage are synthesized from SHED/DPSC studies and regenerative dentistry reviews [12-22]. ALP: alkaline phosphatase, DSPP: dentin sialophosphoprotein, DMP-1: dentin matrix protein 1, RUNX2: runt-related transcription factor 2, CACNA2D2: calcium voltage-gated channel auxiliary subunit alpha2delta 2, TRIO: triple functional domain protein, GAPDH: glyceraldehyde-3-phosphate dehydrogenase, SHED: stem cells from human exfoliated deciduous teeth

Feature	DPSCs	SHED
Tooth source	Permanent teeth	Exfoliated deciduous teeth
Donor age range	Adults	Children (5-12 years)
Proliferation rate	Moderate	Higher
CACNA2D2 basal expression	Low (reference level; present study)	Higher (approximately sevenfold vs DPSCs; present study)
TRIO basal expression	Constitutive, stable (present study)	Constitutive, no significant difference vs DPSCs (ns; present study)
ALP activity/late odontogenic markers	Higher (DSPP, DMP‑1, RUNX2)	Lower
Early mineralization capacity	Moderate [14,17-20]	Stronger (reported) [14,17-20]
Suggested regenerative advantage	Terminal differentiation, long‑term dentin formation [17-19]	Rapid early matrix initiation [14,18-20]

TRIO expression remains stable across conditions

TRIO expression did not differ significantly between undifferentiated DPSCs and odontoblast‑like cells or between DPSCs and SHED (two‑tailed Student’s t‑tests, t(8) = 0.84, p = 0.42, and t(8) = 0.67, p = 0.52, respectively; Figure 2C-2D). Within the limits of this semi‑quantitative approach, the findings support a relatively stable transcript‑level expression pattern for TRIO under the tested conditions.

## Discussion

Principal findings and biological implications

This study provides preliminary wet‑lab evidence that CACNA2D2 is transcriptionally responsive under odontogenic induction conditions in human dental pulp‑derived cells, whereas TRIO transcript abundance remains comparatively unchanged. It also identifies a strong baseline enrichment of CACNA2D2 in SHED relative to DPSCs. Taken together, these data and published observations on SHED and DPSC behavior are summarized in Table 3, highlighting CACNA2D2 enrichment in SHED in the context of their higher proliferative and mineralization tendencies [12,13,16-22]. In that sense, the study serves as an experimental bridge between the previously published Lebanese familial genetics paper and the subsequent in silico mechanistic paper.

Within the constraints of the semi‑quantitative RT‑PCR design, the observed approximate threefold increase in CACNA2D2 expression during odontoblast‑like differentiation is biologically plausible. CACNA2D2 encodes an auxiliary subunit involved in the trafficking and functional regulation of voltage-gated calcium channels, and increased expression during odontogenic commitment could reflect greater demand for calcium-dependent membrane signaling as cells move toward a secretory, matrix-producing phenotype [4,5,7,8]. The present data do not demonstrate that CACNA2D2 directly drives differentiation. Nevertheless, they support the idea that the gene is actively engaged in the molecular environment associated with odontoblast-like maturation [4,6,18-22].

Genetic and stem cell context

This observation is relevant to the prior Lebanese familial tooth agenesis report, which identified a rare CACNA2D2 variant segregating with disease in one family [2]. A candidate gene becomes more persuasive when evidence extends beyond inheritance and computational plausibility to include expression in disease-relevant human cell types [2,3]. The present study does not prove pathogenicity, but it strengthens the biological rationale for continued investigation of CACNA2D2 in tooth development [2-5].

The finding that SHED express much higher basal levels of CACNA2D2 than DPSCs is also noteworthy. Previous work has suggested that SHED and DPSCs are related but not interchangeable stem cell populations, with differences in growth behavior and differentiation tendencies [12-14,16-19]. Higher CACNA2D2 expression in SHED may therefore reflect a broader distinction in calcium‑handling programs between deciduous‑tooth‑ and permanent‑tooth‑derived mesenchymal stem cells [4,5,7,8,18-22]. That interpretation remains speculative, but it creates a useful framework for future comparative functional studies [2,3,18-24].

TRIO interpretation

By contrast, TRIO expression was stable across all tested groups. This negative result should be interpreted carefully because unchanged messenger RNA abundance does not exclude an important biological role [9,25]. TRIO may be regulated through protein abundance, subcellular localization, post-translational modification, or downstream activation of Rho-family signaling rather than through large shifts in transcript levels [9-11,25]. Thus, the present data argue against strong differentiation-dependent transcriptional regulation of TRIO in this model but not against functional relevance in odontogenesis [9-11,21,22,25].

An important strength of the study is that it addresses a narrow and clearly defined unanswered question left by the previous papers. The familial tooth agenesis report established the candidate variants at the clinical‑genetic level, and the in silico paper developed a systems‑level mechanistic hypothesis [2,3]. The present work adds a first layer of wet-laboratory evidence by asking whether CACNA2D2 and TRIO are actually expressed in relevant human dental stem cell models and whether either gene changes during induced odontoblast-like differentiation [2-4,6,12-14,16-18,21,22].

Consequently, the present data should be viewed as an exploratory expression analysis in an odontogenically induced DPSC context, rather than as definitive evidence about CACNA2D2 and TRIO behavior in bona fide, fully characterized odontoblasts [4,6,15,18-22,23].

Limitations and future directions

Several limitations of this study should be acknowledged when interpreting the findings. First, gene expression was assessed using semi‑quantitative RT‑PCR with densitometric analysis rather than RT‑qPCR [15]. This approach is suitable for obtaining an initial, band‑intensity-based overview of transcript behavior. Still, it offers limited quantitative precision and dynamic range, so the reported fold changes should be regarded as approximate, pattern‑level estimates rather than definitive effect sizes. Second, normalization was performed using a single housekeeping gene, GAPDH, instead of a panel of validated reference genes. Reliance on one internal control may leave some residual susceptibility to variation in reference‑gene stability across conditions, which could subtly influence normalized signal intensities. Third, odontoblastic differentiation in the DPSC cultures was inferred from the odontogenic induction protocol and previously published work rather than rigorously validated in the same experimental system. In particular, we did not quantify canonical odontoblast‑associated markers or perform mineralization assays, which are widely considered standard readouts for confirming odontoblastic phenotypes in vitro; for this reason, the term “odontoblast‑like cells” in this manuscript denotes an induced odontogenic context rather than a fully verified terminal phenotype. Fourth, no functional assays were performed to test CACNA2D2 or TRIO activity in dental cells. Specifically, we did not conduct loss‑of‑function or gain‑of‑function experiments (knockdown or overexpression), did not directly modulate calcium channel activity, and did not assess downstream protein expression or signaling readouts, so the present work provides correlative, expression‑level evidence rather than demonstrating a causal role for these genes in odontoblast differentiation or tooth agenesis. Finally, an additional limitation is the modest number of biological donors (n = 5). Although this sample size is acceptable for an exploratory, proof‑of‑concept wet‑lab study and was sufficient to detect robust, directionally consistent trends, it restricts our ability to capture the full extent of donor‑to‑donor biological variability, reduces the precision with which true effect sizes can be estimated, and limits the generalizability of these expression patterns to broader populations of human dental pulp‑derived stem cells.

Taken together, these exploratory findings position CACNA2D2 as a transcriptionally responsive candidate in an odontogenically induced DPSC model and suggest that TRIO expression remains comparatively stable under the same conditions (present study). Future work should therefore follow several complementary directions. Quantitative confirmation by reverse‑transcription quantitative polymerase chain reaction (RT‑qPCR) using multiple validated reference genes, alongside measurement of canonical odontogenic markers and direct mineralization assays in the same cultures, would strengthen confidence in the observed expression patterns [4,6,15,18-22]. Implementing such RT‑qPCR assays and dentin‑related marker/mineralization readouts in the same donor‑matched DPSC and SHED cultures will be an immediate priority in subsequent experimental work and are expected to convert the present band‑intensity‑based findings into fully quantitative, phenotype‑validated data. Functional interrogation of CACNA2D2 and TRIO through knockdown, overexpression, pharmacologic modulation, and calcium‑handling studies (including calcium‑flux and, where feasible, electrophysiologic assays) will be necessary to determine whether these genes are mechanistically involved in odontoblast differentiation and tooth agenesis rather than merely correlated at the transcript level [4,5,7,8,18-22]. Extending these experiments to larger donor cohorts and systematically comparing deciduous‑ and permanent‑tooth‑derived stem cells would also help clarify whether CACNA2D2 contributes to broader differences in calcium‑handling programs between SHED and DPSCs [12-14,16-19,23,24]. In the broader context of familial tooth agenesis, a logical next step will be to integrate such functional differentiation assays with patient‑variant constructs or clustered regularly interspaced short palindromic repeats‑based perturbations to test whether CACNA2D2 or TRIO variants alter odontogenic marker induction and mineralization. Together, these incremental steps, from RT‑qPCR and marker/mineralization validation to functional perturbation of CACNA2D2 and TRIO in larger, systematically phenotyped cohorts, are designed to move this work from an exploratory expression study toward a mechanistically grounded model of odontoblast differentiation and familial tooth agenesis [2-4,9,18-24].

## Conclusions

CACNA2D2 was upregulated during odontoblast‑like differentiation of DPSCs and showed substantially higher basal expression in SHED, whereas TRIO transcript levels remained relatively stable across the tested conditions. Within the limits of a semi‑quantitative single‑housekeeping‑gene design and an odontogenically induced but not fully marker‑validated differentiation model, these findings provide preliminary expression‑level support for a link between CACNA2D2 and an odontogenic DPSC context and extend prior Lebanese clinical‑genetic and in silico observations into an experimental human dental stem‑cell system. Future studies incorporating RT‑qPCR with multiple reference genes, canonical odontoblastic markers, mineralization assays, larger donor cohorts, and targeted functional perturbation of CACNA2D2 and TRIO will be needed to validate these results in bona fide odontoblasts and clarify whether these genes play causal roles in odontoblast differentiation and familial tooth agenesis.
